# Supplementary material for: Estimation of Photosynthetic Induction Is Significantly Affected by Light Environments of Local Leaves and Whole Plants in Oryza Genus
Source: Plants (Basel). 2024 Jun 14;13(12):1646. doi: 10.3390/plants13121646 (PMC11207834; doi:10.3390/plants13121646)
Supplement: Supplementary file 1 [file plants-13-01646-s001.zip › plants-3034000-supplementary.pdf]

Table S1. Steady-state gas exchange parameters across two rice genotypes. All values are means (± SD). A_{steady}, $g_{s,steady}$, $C_{i,steady}$, $g_{m,steady}$ are photosynthetic rate, stomatal conductance, intercellular CO_2 concentration and mesophyll conductance measured at a PPFD of 1500 μmol m^{-2} s^{-1} and CO_2 concentration of 400 μmol mol^{-1}, respectively. V_{cmax} J_{max} Values marked with different letters are significantly different across treatments (P<0.05).

Genotypes	A_{steady} (μmol m^{-2} s^{-1})	$g_{s,steady}$ (mol m^{-2} s^{-1})	$C_{i,steady}$ (μmol mol^{-1})	$g_{m,steady}$ (mol m^{-2} s^{-1})	V_{cmax} (mol m^{-2} s^{-1})	J_{max} (mol m^{-2} s^{-1})
Oryza australiensis	31.0 ± 2.3	0.87 ± 0.14	306 ± 10	0.18 ± 0.02	137 ± 14	169 ± 11
Oryza officinalis	19.2 ± 0.4	0.41 ± 0.05	294 ± 10	0.11 ± 0.01	89.9 ± 8.5	138 ± 8

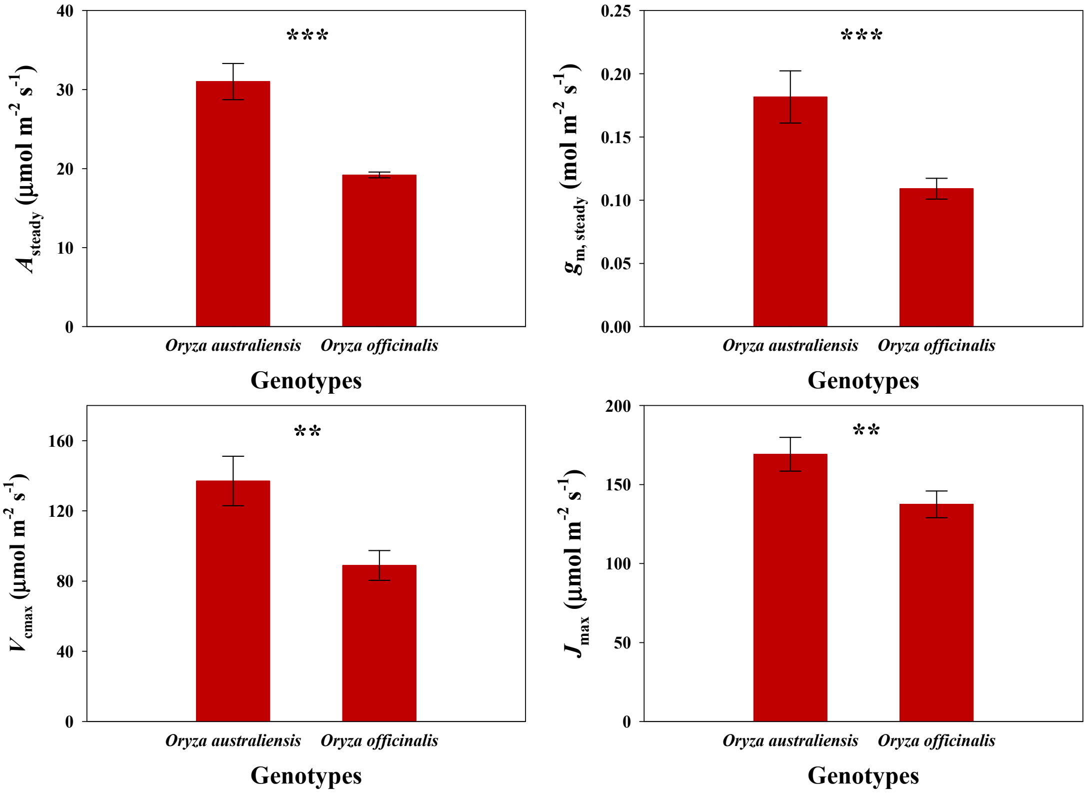

Figure S1. Steady-state gas exchange parameters across two rice genotypes. A_{steady} and $g_{m,steady}$ are photosynthetic rate and mesophyll conductance measured at a PPFD of 1500 μmol m^{-2} s^{-1} and CO_2 concentration of 400 μmol mol^{-1}, respectively. V_{cmax} and J_{max} are maximum velocity of Rubisco for carboxylation and maximum rate of electron transport, respectively. All values are means (± SD) of four measurements. Significance

levels are indicated by *** and ** that indicate P<0.001, and P<0.01 respectively
